# Challenges Experienced by Novice Undergraduate Nursing Students in Their Clinical Learning Environment: A Qualitative Study

**DOI:** 10.7759/cureus.97322

**Published:** 2025-11-20

**Authors:** Prathibha Rani S K

**Affiliations:** 1 Nursing Education and Administration, Government College of Nursing, Thiruvananthapuram, IND

**Keywords:** challenges, clinical learning, clinical learning environment, nursing education, undergraduate nursing students

## Abstract

Background

Clinical learning is the core and integral part of nursing education, which transforms a student nurse into a vibrant and competent professional nurse who is capable of rendering comprehensive quality care to the demanding society. Clinical placement is the cornerstone of nursing education. However, for first-year students, transitioning from classroom learning to real clinical environments poses significant challenges.

Aim

The study aimed to provide an in-depth exploration of the challenges experienced by novice undergraduate nursing students in their clinical learning environment.

Methods

A hermeneutic phenomenological qualitative research design was employed for the study. Data were collected from 32 first-year undergraduate nursing students through in-depth interviews (IDIs) and focus group discussions (FGDs). The qualitative data were analysed by thematic analysis using Braun and Clarke’s six-step method.

Results

The study findings revealed that novice nursing students experience challenges in their clinical learning environment, which includes emotional struggles in clinical exposure, theory-practice gap, environmental and infrastructural challenges, role ambiguity and lack of confidence, academic pressure and career disappointment, communication and confidence challenges, and workload and physical strain.

Conclusion

First-year undergraduate nursing students encounter multifaceted challenges that impact their learning and well-being. Structured mentorship, better orientation, and systemic and unwavering support are necessary to enhance positivity in the clinical learning experience of these students.

## Introduction

Clinical learning is a vital and integral component of nursing education, serving as a bridge between theoretical knowledge and practical skills required in patient care. Clinical placements for nursing students are important in many respects and are characterized as an irreplaceable component of nursing education. From a legal perspective, practice in clinical settings is a requirement to ensure fitness to practice as a nurse. From an educational perspective, the clinical placement is the venue where skills, knowledge, and attitudes developed in the theoretical part of the curriculum are applied, developed, and integrated [1]. For first-year nursing students, this transition is often overwhelming due to their lack of previous exposure to real clinical situations.

Clinical practice provides students with the opportunity to apply their structured cognitive, affective, and psychomotor gains from classrooms to real-life [2]. This experiential learning has a pivotal role during the first year, as it marks the beginning of a student's professional identity formation and skill development. However, first-year nursing students frequently encounter a wide array of challenges as they navigate the complex shift from structured classroom learning to the dynamic and often unpredictable clinical environment [2,3]. These challenges may include feelings of anxiety and self-doubt, difficulty in communication with patients and healthcare staff, lack of confidence in performing clinical skills, and struggles in integrating theory with practice. Understanding these initial hurdles is crucial for educators and institutions to design supportive strategies that ease the transition and enhance clinical learning outcomes [4].

Nursing education in Kerala has advanced to global standards, and there are about 250 nursing institutions offering diploma and basic nursing programs. More than 2500 students are getting admitted to these courses every year. The first year nursing students will be exposed to various medical and surgical wards to meet their learning objectives, and they will spend more than three to four months they spent in these clinical areas. Being entered into a totally unaware and unexposed place will definitely create apprehension and uncertainty in their minds.

Multiple studies have highlighted that first-year nursing students commonly experience anxiety, stress, and fear during their initial clinical placements [1,3,4,5,6]. These emotional responses are often triggered by unfamiliar hospital settings, communication barriers, complex patient conditions, and fear of making mistakes [7]. Moreover, the discrepancy between what is taught in simulation labs and what is encountered in actual practice creates a significant theory-practice gap, impeding their confidence and performance.

A conducive clinical learning environment (CLE) includes adequate supervision, positive interpersonal relationships, clearly defined student roles, and opportunities for hands-on learning [8]. However, in many settings, high student-to-faculty ratios, overcrowded wards, insufficient equipment, and a lack of structured orientation programs exacerbate the challenges faced by novice students [9,10].

Background of the study

Several international and national studies have investigated the difficulties nursing students encounter in clinical settings. Chan introduced the Clinical Learning Environment Inventory (CLEI) to assess student perceptions and identified supervision and student-teacher relationships as key influencing factors [3]. Sundler et al. revealed that poor organization of clinical supervision, like inadequate clinical supervisors, limited interaction between supervisor and student, etc., often hinders learning and causes frustration [4]. Jamshidi et al. emphasized that unclear instructions and fear of making mistakes were primary stressors among novice students [5]

Moreover, Henderson et al. suggested that structured clinical support programs and mentorship improve student satisfaction and reduce anxiety [6]. Al-Gharibi et al., in a systematic review, found consistent evidence that first-year students struggle with their own role confusion in the clinical area, lack of feedback, and difficulty in patient communication [8].

A study by Varghese et al. identified various challenges faced by nursing students in the CLE, primarily focusing on factors like inadequate facilities, fear of making mistakes, and a high number of students in the clinical area [11]. A descriptive phenomenological study conducted by Damodaran and Kandasamy in Kerala revealed that the nursing students experienced anxiety, lack of confidence, and variation in clinical guidance and positive supervision enhanced learning satisfaction [12].

However, the CLE of fresh nursing students is the least explored area in Kerala. Further, the researcher, through her own experience of more than 15 years with novice nursing students in the clinical area, identified a lot of struggles faced by these students to immerse themselves in meeting their learning outcomes, and was motivated to further explore this topic. The purpose of the present study was to explore the challenges experienced by the new nursing students by interpreting their lived experiences in the CLE.

Objectives

The objectives of this study are to explore the lived experiences of novice nursing students during clinical postings and to interpret the challenges faced by novice nursing students in CLEs.

## Materials and methods

Methodology

Design

A hermeneutic phenomenological qualitative research design was adopted for the study. Hermeneutic phenomenological qualitative research designs combine phenomenology's focus on lived experience with hermeneutics’ emphasis on interpretation to understand a phenomenon through the lens of both the participant's experience and the researcher's interpretation.

Sample

Thirty-two first-year undergraduate nursing students who have completed their first semester clinical posting and are willing to participate in the study were selected using a purposive sampling technique from three nursing educational institutions in Kerala. Informed consent was obtained from all the participants.

Data Collection Method

Data were collected using in-depth interviews (IDIs) and focus group discussions (FGDs). Six FGDs were conducted with four to five students in one group. Five IDIs among students were conducted to get more detailed data. The number of FGDs and IDIs was determined by redundancy of data. A semi-structured interview guide was used to guide the interviews. The data collection process was done in a peaceful environment of the concerned educational institutions. The interviews included open-ended questions about each participant’s clinical learning experience, the difficulties they face in their clinical setting, and their suggestions for overcoming the difficulties. Topics included emotional experiences, communication issues, supervision, theory-practice alignment, workload, and environmental challenges. Each interview lasts for about 30-40 minutes. All audio-recorded sessions were transcribed verbatim immediately after each data collection activity to ensure accuracy and completeness of participants’ narratives. Nonverbal cues such as pauses, laughter, or emotional expressions were noted in the transcripts to preserve the contextual meaning of the participants’ responses. The transcription process was conducted manually by the researcher to maintain close engagement with the data and to begin the process of immersion, which is vital in phenomenological analysis. Each transcript was reviewed alongside the audio recordings to verify the accuracy of the transcribed text and to correct any discrepancies.

Since some interviews and discussions were conducted in the participants’ native language, translation into English was necessary for analysis and reporting. A forward-backward translation process was used to preserve the authenticity and intended meaning of participants’ statements. The initial translation from the local language to English was carried out by a bilingual researcher familiar with nursing education terminology. A second independent translator then performed a back-translation into the original language. Any differences were discussed and reconciled to ensure semantic and conceptual equivalence. This rigorous translation process minimized the loss of meaning and cultural nuances.

Data triangulation was employed to enhance the trustworthiness and credibility of the study findings. In this research, triangulation involved the integration of data obtained from two sources - FGDs and IDIs - to gain a more comprehensive understanding of the challenges experienced by novice nursing students in their CLE. FGDs provided a collective perspective, capturing shared experiences, group dynamics, and commonly faced issues among students, while IDIs allowed for deeper exploration of individual experiences, emotions, and personal reflections that might not emerge in a group setting. The combination of these methods enabled the researcher to compare and contrast insights across datasets, identify converging themes, and explore any divergences in perception or experience.

Analysis

Thematic analysis was employed in the study to analyze interview data and to obtain an in-depth understanding of the phenomenon under investigation. This method involves systematically identifying, organizing, and examining patterns (themes) within the data. The analysis followed Braun and Clarke’s six-phase framework, which includes: (1) familiarization with the data, (2) generation of initial codes, (3) searching for themes and subthemes, (4) reviewing themes, (5) defining and naming themes, and (6) producing the final report. This approach enabled a comprehensive exploration of participants’ experiences and perspectives.

## Results

Findings

A total of 32 first-year undergraduate nursing students participated in the present study.

Table 1 presents the demographic characteristics of the participants. A total of 22 participants were female and 10 were male, with an average age of 20.64 ± 2.28. Thematic analysis of the IDI and FGD transcripts led to the emergence of seven major themes supported by codes and direct participant quotes, as shown in Table 2.

**Table 1:** Demographic variables of nursing students

Demographic Variable	Category	Frequency (n)	Percentage (%)
Gender	Male	12	37.5
	Female	20	62.5
Age in Years	18	10	31.3
	19	20	62.5
	20	2	6.2

**Table 2:** Overview of main themes, sub-themes, and codes from thematic analysis

Theme	Sub-themes	Codes	Supportive Quotes
1. Emotional Struggles in Clinical Exposure	a. Sense of inferiority. b. Emotional distress.	Embarrassment, shame, sadness, fear	“I feel embarrassed to be in the ward holding the tray to take care of patients. Everyone seems to be laughing at me.” “I feel sad while watching MBBS students in the ward. My wish was also to become a doctor.”
2. Theory-Practice Gap	a. Discrepancy between learning and practice. b. Lack of clinical resources.	Skill discrepancy; lack of equipment	“I am able to do things in the classroom and lab, but in the ward the articles are limited.” “Clinicals were beyond my expectations.”
3. Environmental and Infrastructural Challenges	a. Overcrowded clinical settings. b. Poor hospital layout and navigation.	Overcrowded wards; lack of signage	“The ward is overcrowded, and I don’t know where to focus.” “There are no proper signboards in the hospital. I feel shy when I can’t direct someone properly.”
4. Role Ambiguity and Lack of Confidence	a. Unclear clinical roles. b. Lack of guidance from supervisors.	Unclear roles; fear of mistakes	“We don’t know exactly what we are expected to do.” “Students and patients are many, and only one supervisor. Difficult to get attention.”
5. Academic Pressure and Career Disappointment	a. Unmet career aspirations. b. Academic overload.	MBBS rejection; assignment burden	“I tried many times for MBBS but failed.” “Too many assignments to complete in a short time, so I feel anxious.”
6. Communication and Confidence Challenges	a. Anxiety in patient interaction. b. Unrealistic expectations.	Fear of speaking; role confusion	“I feel shy and embarrassed to talk with patients.” “Relatives ask about ICUs and labs like we know everything.”
7. Workload and Physical Strain	a. Physical fatigue. b. Inadequate supervision.	Long hours; fatigue; insufficient support	“Standing for long hours is really horrible to adjust.” “Only one supervisor for all of us. Hard to get help.”

The main themes derived are emotional struggles in clinical exposure, theory-practice gap, environmental and infrastructure challenges, role ambiguity and lack of confidence, academic pressure, communication and confidence challenges, and workload and physical strain.

Emotional Struggles in Clinical Exposure

The initial transition into the clinical setting was described by many participants as emotionally overwhelming. Students expressed a range of intense emotions such as embarrassment, shame, sadness, fear, and a general sense of emotional unpreparedness. These feelings were often triggered by the perceived scrutiny from patients, caregivers, and healthcare professionals in the ward.

One participant shared the discomfort of performing basic patient care tasks in a public setting, stating:

“I feel embarrassed to be in the ward holding the tray to take care of patients. Everyone seems to be looking at me.” (Participant 4)

This sense of being observed contributed to a feeling of a lack of confidence in oneself. Several participants echoed this sentiment, reporting that they felt judged or out of place in the hospital environment. These emotional responses appeared to stem not only from the newness of the environment but also from internalized comparisons with other healthcare students, such as medical peers, who appeared more confident or socially accepted.

The data suggest that first-year nursing students often enter the clinical space without adequate emotional preparation, which can lead to insecurity and hesitation. These emotional barriers may impede their ability to learn effectively and interact comfortably with the clinical team. Addressing emotional preparedness and offering psychological support early in the clinical education process may help ease this transition and improve student engagement in practice.

Theory-Practice Gap

A prominent challenge reported by participants was the disconnect between theoretical learning and practical application in the clinical environment. Despite performing confidently in classroom demonstrations and nursing foundation labs, students experienced uncertainty and confusion when faced with the realities of hospital wards. The gap was primarily attributed to a lack of essential equipment, unfamiliar ward routines, and the dynamic, unpredictable nature of real-life patient care.

One student reflected on this experience, stating:

“I am able to do things in the classroom and simulation lab, but in the ward, the articles are limited, and I don’t know where to focus.” (Participant 7)

This statement underscores the difficulties students face when expected to translate structured, idealized procedures from the lab into less controlled clinical situations. Several participants noted that the lack of familiarity with the ward environment and the limited availability of necessary items hindered their ability to perform skills confidently.

The disconnect between academic preparation and clinical expectations often led to feelings of inadequacy and hesitation. Many students reported that the lab-based training did not fully prepare them for managing patient care amidst time constraints, incomplete resources, and multitasking demands. These findings point to the need for a more integrated clinical teaching approach-bridging simulation and ward reality-to better support students in developing practical competence and clinical judgment.

Environmental and Infrastructural Challenges

Environmental and infrastructural barriers within the clinical setting emerged as significant stressors for nursing students. Participants described the hospital environment as physically overwhelming and poorly organized, which hindered their ability to orient themselves, perform tasks efficiently, and focus on learning. Overcrowded wards, lack of clear signage, and a chaotic atmosphere contributed to a sense of disorientation and frustration.

One student shared their experience:

“The ward is overcrowded and confusing. There are no proper signboards, and relatives keep asking us directions as if we know everything.” (Participant 9)

This reflection illustrates the strain of being placed in unfamiliar and high-pressure environments where students were not only expected to learn but also to respond to patient relatives and navigate logistical challenges. For many, this added a layer of emotional and cognitive burden that distracted from their primary learning objectives.

The absence of structured orientation and clear navigation within the hospital setting often made students feel lost and anxious, further impacting their confidence and ability to engage with patient care. These findings highlight the importance of hospital-based orientation programs and infrastructure improvements (e.g., signboards, student-friendly maps) to enhance the clinical learning experience and reduce unnecessary stress for novice learners.

Role Ambiguity and Lack of Confidence

First-year nursing students often encounter uncertainty regarding their roles and responsibilities within the clinical environment. This ambiguity results in hesitation, confusion, and a decline in self-confidence. In the absence of clearly outlined expectations, students may find it difficult to define their scope of practice, leading them to frequently question their own actions. Such uncertainty fosters a fear of judgment or criticism from patients, clinical staff, and peers, ultimately limiting their ability to participate fully and develop confidence in clinical practice.

One participant candidly expressed:

“We don’t know exactly what we are expected to do, and I am scared of making mistakes in front of everyone.” (Participant 6, 10)

This quote reflects the internal struggle faced by many students who feel unprepared to navigate clinical responsibilities independently. The fear of making mistakes, especially in a public and professional environment, significantly affected their willingness to participate actively in patient care.

Uncertainty about clinical duties often led students to adopt a passive role, relying heavily on instructors or senior staff for direction. This dependency further inhibited the development of clinical autonomy and decision-making skills. The findings suggest that the presence of structured orientation, clear task delegation, and mentorship could help reduce role ambiguity and boost students’ confidence during their early clinical exposure.

Academic Pressure and Career Disappointment

For several students, the CLE was compounded by deeper personal and academic challenges, particularly those stemming from unfulfilled career aspirations and overwhelming academic demands. Many participants shared feelings of disappointment after failing to gain admission into the MBBS program - a dream they had long nurtured. Entering nursing education as a secondary option created emotional distress, which was further exacerbated by academic overload.

One student shared:

“My dream was to become a doctor. I tried many times for MBBS but failed. Now the number of assignments is too much; I feel very anxious.” (Participant 3)

This narrative reveals a layered experience of grief and anxiety stemming from both perceived academic failure and current academic pressure. Students described struggling to find motivation and identity within nursing, especially when it was not their first choice. The emotional residue of career disappointment, combined with heavy coursework, contributed to stress, poor focus, and reduced engagement in both theory and clinical sessions.

These findings underscore the need for emotional and academic support mechanisms within Nursing programs. Counseling services, career guidance, and academic mentoring could help students reconcile with their career path and manage the academic expectations placed upon them more effectively.

Communication and Confidence Challenges

Effective communication is essential in clinical learning, yet many students reported feeling hesitant and insecure when interacting with patients and caregivers. A lack of confidence in their communication skills, compounded by limited clinical exposure and uncertainty about their roles, made verbal interactions a source of stress rather than learning.

One participant explained:

“I feel shy and embarrassed to talk with patients and caregivers. I don’t know what to say or how to respond.” (Participant 5)

This statement reflects a common fear among novice students: anxiety about saying the wrong thing, not knowing how to initiate conversations, or being judged for their inexperience. Such apprehensions often led to students avoiding patient interactions altogether, further limiting their learning and integration into the clinical team.

Role confusion also contributed to this challenge. Without clear guidance on how to approach patients or what was expected in terms of communication, students were left feeling unsure and passive. This highlights the need for structured communication training, supervised roleplaying, and supportive feedback to help students build interpersonal confidence and develop a professional bedside manner.

Workload and Physical Strain

Clinical placements were described as physically demanding for first-year nursing students. Participants reported long hours of standing, physical fatigue, and a lack of adequate rest periods.

One participant shared:

“We stand for long hours, there is no resting rooms in the clinical area at least to sit for a while when we are sick or tired….. deadly exhausted during the end of the day of clinical posting” (Participants 2, 7, 10)

This quote reflects the physical strain of clinical duties. The clinical environment is not able to meet the needs and demands of students. Physical exhaustion due to prolonged standing can lead to emotional fatigue, which can further hinder their learning and attitude.

Addressing this issue may require restructuring clinical schedules by slowly increasing the hours of duty for fresh students. Lecture class rooms in the clinical area can be used for short spans of clinical teaching and discussions.

## Discussion

This study explored the challenges experienced by first-year nursing students in their CLE. The qualitative data revealed seven interrelated themes that reflect the emotional, academic, structural, and interpersonal difficulties faced by novice learners. This part discusses each theme in detail, relating the findings to previous research and exploring their implications for nursing education and clinical practice.

Emotional struggles in clinical exposure

Emotional responses, such as embarrassment, fear, and shame, emerged as a dominant theme in this study. Many students reported feeling exposed and judged while performing tasks in the ward, particularly during interactions with patients, bystanders, and other healthcare professionals. These emotional reactions can be attributed to a sudden transition from a protected classroom setting to the unpredictable clinical environment, where students are expected to perform confidently despite their inexperience.

Previous studies have similarly reported that emotional distress is common among novice nursing students during clinical exposure. According to Sharif and Masoumi, emotional discomfort during early clinical experiences can hinder skill application and contribute to avoidance behaviors [10]. Moreover, students with low emotional resilience are more likely to disengage from clinical opportunities, thereby limiting their professional growth. To address this issue, nursing curricula must include psychological preparedness training and build emotional intelligence through reflective practice, peer debriefing, and mentorship.

Theory-practice gap

Participants consistently voiced frustration over the disconnect between what is taught in classrooms and labs and what is experienced in real clinical settings. While students demonstrated proficiency in simulated environments, they struggled to perform confidently in hospital wards due to a lack of materials, procedural deviations, and contextual differences.

This gap between theory and practice is a well-documented challenge in nursing education. Benner’s model of novice-to-expert emphasizes that students begin as novices who require repetitive, guided practice in real contexts to build competence [13]. However, when clinical settings fail to provide adequate opportunities or resources, students cannot reinforce their theoretical learning. Jamshidi et al. also found that insufficient alignment between classroom instruction and clinical expectations leads to anxiety, skill hesitation, and loss of professional identity [14].

To mitigate this, nursing programs should emphasize early clinical exposure, increase collaboration between academic and clinical faculty, and adopt case-based learning that mirrors actual hospital situations.

Environmental and infrastructural challenges

The clinical environment itself was found to be overwhelming for many students. Overcrowded wards, absence of proper signage, and chaotic patient traffic created confusion, stress, and disorientation. Students felt lost within the hospital premises, often being asked for directions they themselves were unsure of, leading to discomfort and cognitive overload.

This is consistent with findings from Ullah et al., who reported that unfamiliar and poorly organized hospital environments contribute to student stress and hinder clinical focus [15]. The physical setting is not merely a backdrop for learning-it actively influences student confidence, orientation, and safety. Inadequate infrastructure also increases reliance on instructors for basic navigation and task performance, further straining already limited faculty resources.

Institutional solutions could include structured clinical orientation, designated student support points, improved hospital signage, and pre-placement walkthroughs to familiarize students with clinical layouts and ward routines.

Role ambiguity and lack of confidence

A significant number of participants reported confusion about their clinical duties and the scope of practice. They were unsure of what was expected from them, which created fear of making mistakes in front of others. This lack of role clarity led to low self-confidence and reduced initiative, causing some students to adopt passive roles during clinical postings.

These findings echo the results of Papastavrou et al., who noted that unclear roles lead to role conflict, internalized stress, and missed learning opportunities [16]. First-year students are especially vulnerable to role confusion as they lack the contextual understanding of professional hierarchies, task boundaries, and clinical protocols.

To address this issue, nurse educators and clinical instructors must clearly define student roles, provide daily objectives, and encourage open dialogue to clarify doubts. Structured clinical handbooks and task checklists could also enhance students’ understanding of responsibilities.

Academic pressure and career disappointment

Some participants shared deep personal disappointment about not gaining admission to medical school (MBBS) and described entering nursing as a compromise. These unresolved aspirations, coupled with heavy academic workloads and frequent assignments, contributed to feelings of frustration and academic anxiety. Students expressed difficulty staying motivated and mentally invested in their nursing education.

This theme aligns with research by Alzayyat and Al-Gamal, who found that unmet career expectations, when combined with high academic pressure, often result in psychological distress and poor academic performance among nursing students [17]. These emotional undercurrents can diminish professional identity formation and lead to a lack of engagement in clinical practice.

Counseling services, peer mentorship, and personalized academic advising are essential strategies to support students in reconciling their career paths. Positive role modelling by faculty and exposure to the rewarding aspects of nursing may also help students shift their mindset and embrace their professional identity.

Communication and confidence challenges

Fear of speaking with patients and caregivers, along with uncertainty about appropriate language and responses, emerged as a key challenge. Many students felt shy, embarrassed, and unsure how to initiate or sustain clinical conversations. The fear of being judged or misunderstood led to communication avoidance, which in turn limited learning opportunities and patient interaction.

Lavoie-Tremblay et al. found that communication anxiety is a common barrier to effective clinical learning among student nurses, especially in the early stages [18]. Communication competence is not innate - it develops through exposure, feedback, and guided practice. Lack of communication skills not only affects patient safety but also undermines interprofessional collaboration and student confidence.

Nursing programs should incorporate structured communication training, including simulation, standardized patient encounters, and roleplays. Providing scripts for common clinical scenarios and feedback from instructors can help students gain fluency in professional dialogue.

Workload and physical strain

Physical exhaustion due to long hours of standing and minimal rest was a major concern. Additionally, limited clinical supervision resulted in students competing for attention, thereby reducing personalized feedback and guidance. Participants reported feeling drained and overlooked, which demotivated them from participating fully in clinical tasks.

Reeves et al. have emphasized the link between physical strain, lack of supervision, and reduced clinical competence [19]. When students are physically fatigued, their ability to concentrate, retain information, and engage actively in care processes diminishes. Moreover, inadequate instructor-to-student ratios can lead to missed learning opportunities and unsafe practice.

Clinical schedules should be adjusted to include sufficient rest, rotation-based task assignments, and a maximum cap on student-to-instructor ratios. Periodic physical health assessments and wellness activities may further promote student endurance and enthusiasm.

Limitations of the study

The findings are based on a relatively small sample size drawn from three institutions, which restricts the generalization of the results. The use of FGDs may have introduced social desirability bias or group conformity, where participants might have withheld sensitive or negative experiences in the presence of their peers. Although IDIs were conducted to minimize this limitation, certain aspects of participants’ emotional experiences could still remain unspoken. Since the study relied on self-reported narratives, the data reflect participants’ recollections and perceptions, which may be affected by memory, selective recall, or personal interpretation. Direct observation of clinical experiences could have provided additional validation, but it was beyond the scope of this study. Time constraints limited data collection to a particular period, thereby preventing exploration of how students’ perceptions might evolve with increased clinical exposure.

Recommendations

Structured preclinical orientation programs should be developed with an emphasis on emotional preparedness to help students cope with anxiety, stress, and the transition from classroom to clinical practice. Establishing an effective mentorship system, including both faculty guidance and peer support networks, can provide students with continuous encouragement and a safe space to discuss challenges. Communication and interpersonal skill training should be given more priority to enhance students’ confidence in interacting with patients, families, and healthcare professionals. Maintaining an adequate faculty-to-student ratio in clinical areas is essential to ensure close supervision and timely feedback. Future research should focus on evaluating the effectiveness of these strategies across different stages of the nursing program.

## Conclusions

This qualitative study explored the lived experiences of first-year nursing students as they navigated their initial exposure to clinical practice. Using thematic analysis of IDIs and FGDs, the study uncovered seven major themes that reflect the multifaceted challenges fresh nursing students face in real-world clinical environments. These include emotional struggles, a gap between theory and practice, environmental and infrastructural barriers, role ambiguity, academic pressure, communication difficulties, and physical strain. The findings reveal that clinical learning for novice nursing students is not only about skill acquisition but also about emotional adaptation, professional identity formation, and coping with complex systemic and contextual factors. Students enter the clinical setting with enthusiasm but often encounter emotional discomfort, confusion, and a lack of confidence-especially when clinical settings do not align with their academic preparation or personal expectations.

Ultimately, the quality of clinical learning experiences during the formative years plays a crucial role in shaping nursing students’ professional identity, clinical competence, and long-term career satisfaction. The challenges identified in this study, ranging from emotional struggles and communication barriers to workload pressures and limited supervision, highlight the need for targeted interventions. Addressing these issues through supportive clinical teaching models, enhanced mentorship, and resource allocation can foster a more enriching and empowering CLE. Such efforts are vital in fostering the development of confident, competent, and compassionate nurses to meet the demands of the modern healthcare system.
